# Comparative Analysis of Novel Inflammatory and Nutritional Indices (m-HALP, HALP, SII, SIRI) for Risk Stratification in Septic Patients: A Retrospective Cohort Study

**DOI:** 10.3390/life16081327

**Published:** 2026-08-13

**Authors:** Salih Kocaoğlu, Tufan Alatlı, Deniz Sığırlı, Selman Gümüş

**Affiliations:** 1 Department of Emergency Medicine, Faculty of Medicine, Balıkesir University, Balıkesir 10000, Türkiye; drtufanalatli@gmail.com (T.A.); dr.selmangumus@gmail.com (S.G.); 2 Department of Statistics and Business Analytics, United Arab Emirates University, Al Ain 15551, United Arab Emirates; sigirli@uludag.edu.tr; 3 Department of Biostatistics, Faculty of Medicine, Bursa Uludağ University, Bursa 16000, Türkiye

**Keywords:** sepsis, m-HALP score, SII, SIRI, mortality

## Abstract

**Background:** Reliable biomarkers integrating inflammatory and nutritional status are crucial for sepsis risk stratification. While indices like the Systemic Immune-Inflammation Index (SII) and Systemic Inflammation Response Index (SIRI) are widely studied, the prognostic value of the modified Hemoglobin, Albumin, Lymphocyte, and Platelet (m-HALP) score in sepsis remains unclear. This study aimed to compare the prognostic accuracy of m-HALP, HALP, SII, and SIRI for 90-day mortality in patients diagnosed with sepsis in the emergency department. **Materials and Methods:** This single-center, retrospective observational study included 188 adult patients diagnosed with sepsis according to the Surviving Sepsis Campaign guidelines. Routine hemogram and biochemistry parameters obtained at admission were used to calculate SII, SIRI, HALP, and m-HALP scores. The primary endpoint was 90-day mortality, and secondary endpoints included 7-day mortality, ICU admission, and mechanical ventilation requirement. The discriminative performance of the indices was assessed using Receiver Operating Characteristic (ROC) curve analysis. **Results:** The median age of the cohort was 75.5 years, and the 90-day mortality rate was 49.5%. Among the evaluated indices, only the lower m-HALP scores demonstrated a statistically significant association with 90-day mortality in the overall cohort (AUC: 0.590, 95% CI: 0.509–0.671, *p* = 0.030). In the pneumosepsis subgroup, the prognostic performance of m-HALP was numerically higher (AUC: 0.667, 95% CI: 0.548–0.785, *p* = 0.006) with a sensitivity of 57.4% and specificity of 76.7% at a cut-off value of 522.11. Conversely, standard HALP, SII, and SIRI did not show statistically significant predictive value for 90-day mortality (*p* > 0.05). None of the indices successfully predicted 7-day mortality, ICU admission, or mechanical ventilation requirement. **Conclusions:** While m-HALP showed a statistically significant association with 90-day mortality, particularly in the pneumosepsis subgroup, its overall discriminative ability was poor-to-modest (overall AUC 0.590; pneumosepsis AUC 0.667). Consequently, m-HALP lacks sufficient accuracy for standalone clinical decision-making, rendering these findings strictly exploratory and hypothesis-generating. Future prospective, multicenter studies are needed to validate this independent association.

## 1. Introduction

Sepsis is defined as life-threatening organ dysfunction caused by a dysregulated host response to infection and remains a leading cause of morbidity and mortality in intensive care units (ICU) worldwide [1,2]. Despite advances in diagnostic and treatment protocols (Surviving Sepsis Campaign), sepsis-related mortality rates remain unacceptably high, accounting for approximately 20% of the global health burden [3,4]. Particularly in the elderly population, the presence of immunosenescence and comorbidities further complicates clinical management and increases the need for reliable biomarkers capable of early risk stratification [5].

The pathophysiology of sepsis is characterized not only by an imbalance between pro-inflammatory and anti-inflammatory responses but also by severe metabolic disturbances and malnutrition [6]. Although traditional inflammatory markers such as C-reactive protein (CRP) and procalcitonin (PCT) are widely used in clinical practice, these parameters may not always provide holistic information regarding the patient’s nutritional status or cellular immune response [7]. Among existing prognostic models, the Sequential Organ Failure Assessment (SOFA) score is not suitable for initial triage due to its complex calculation requirements. Furthermore, the quick SOFA (qSOFA) score, previously recommended for bedside screening, is no longer suggested as a standalone screening tool in the 2021 Surviving Sepsis Campaign guidelines due to its low sensitivity [1,8]. Therefore, in recent years, there has been increasing interest in cost-effective and easily accessible composite indices derived from complete blood count parameters that can simultaneously reflect both systemic inflammation and nutritional status.

The Systemic Immune-Inflammation Index (SII) and Systemic Inflammation Response Index (SIRI) are markers based on combinations of neutrophil, lymphocyte, monocyte, and platelet counts, with proven prognostic value in various clinical scenarios ranging from oncological surgeries to cardiovascular diseases [9,10,11]. While some recent studies in septic patients suggest that elevated SII and SIRI values are associated with poor prognosis, the consistency of these indices across different infection sources (pneumosepsis, urosepsis, etc.) and their impact on long-term mortality remain a subject of debate [12,13,14].

On the other hand, the HALP score, comprising Hemoglobin, Albumin, Lymphocyte, and Platelet levels, and the m-HALP score are newer indices that integrate both the patient’s immuno-inflammatory status and nutritional reserve. First defined by Chen et al. as a prognostic marker in malignancies, the HALP score was subsequently optimized by Kocaoglu et al. and introduced into the literature as the ‘modified HALP (m-HALP),’ where lower scores were shown to be significantly associated with mortality in acute decompensated heart failure [15,16].

The primary aim of this study is to determine the prognostic value of the HALP, m-HALP, SII, and SIRI indices in patients diagnosed with sepsis in the emergency department. In this context, we aimed to investigate the relationship of these indices with clinical severity scores and mortality, particularly focusing on long-term (90-day) mortality as well as short-term (7-day) mortality. Additionally, the study aims to demonstrate the contribution of these composite biomarkers derived from routine hemogram parameters to clinical decision-making processes and to reveal potential differences in prognostic performance based on the focus of infection.

## 2. Methods

### 2.1. Study Design and Ethical Approval

This single-center, retrospective observational study included patients aged 18 years and older who presented to the Balıkesir University Faculty of Medicine Emergency Department and were diagnosed with sepsis. A total of 188 sepsis patients were included in the study. Ethical approval was obtained from the Balıkesir University Health Sciences Non-Interventional Research Ethics Committee (Decision No: 2025/227, Date: 16 May 2025). Due to the retrospective design of the study, the requirement for informed consent was waived by the ethics committee in accordance with the principles of the Declaration of Helsinki.

### 2.2. Patient Selection and Data Collection

**Figure 1:**
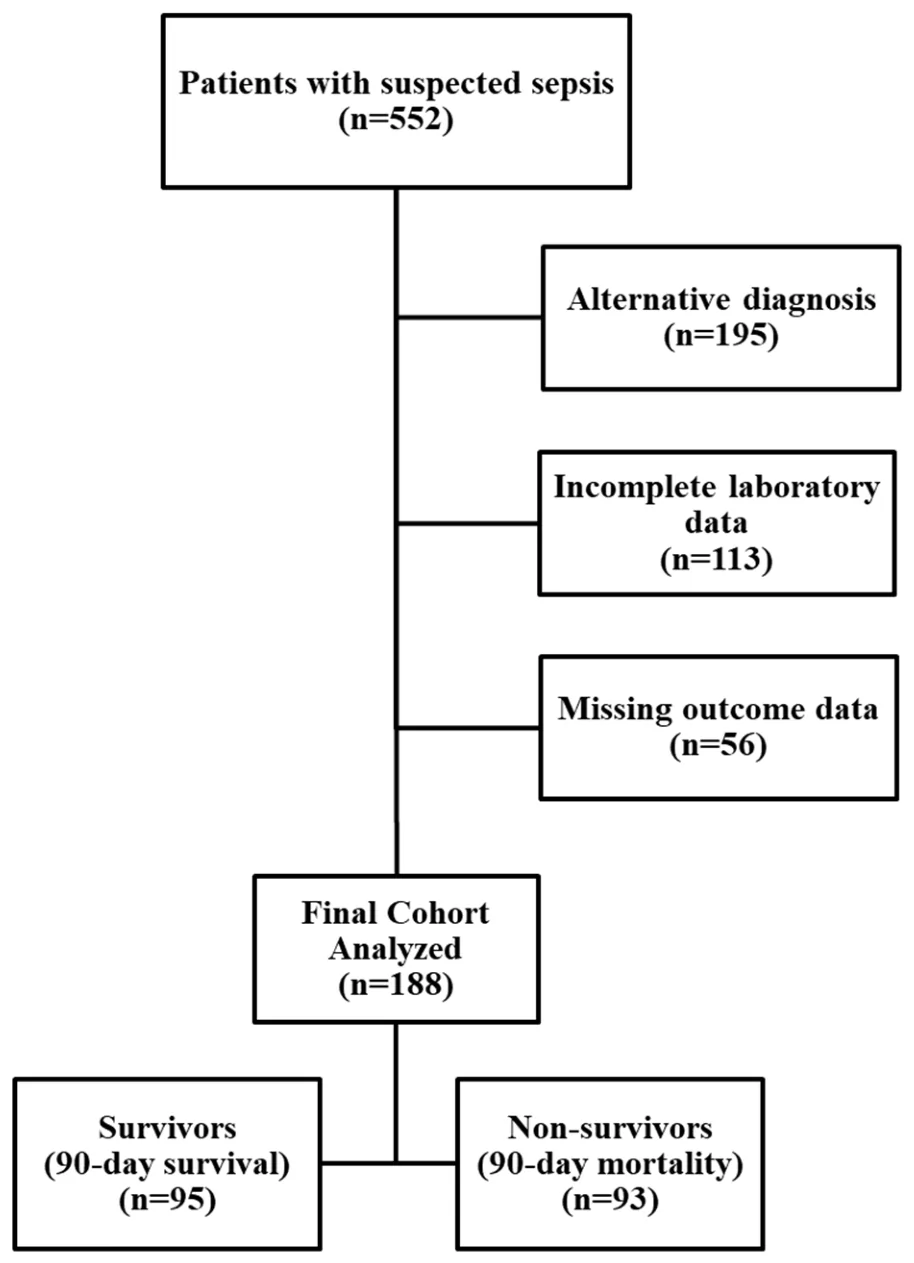
Patient selection flow diagram.

A total of 552 patients initially admitted to the emergency department with suspected sepsis according to the “Surviving Sepsis Campaign” guidelines were screened for the study. Following detailed clinical evaluation and consultation with infectious disease specialists, 195 patients who received alternative non-sepsis diagnoses (e.g., acute heart failure, COPD exacerbation, pulmonary embolism, acute myocardial infarction) were excluded from the study. From the remaining cohort, 113 patients with missing mandatory laboratory parameters (especially albumin) required for calculating the investigated indices, and 56 patients with inaccessible 90-day survival follow-up data (the primary endpoint) were excluded. As a result of these strict exclusion criteria, the remaining 188 patients were included in the final analysis. The included patients were classified according to the source of infection (pneumosepsis, urosepsis, cholangiosepsis, etc.). The patient selection flow diagram is presented in Figure 1.

Data were obtained retrospectively from the hospital’s electronic record system and patient files. The following demographic and clinical parameters were recorded: age, gender, source of infection, ICU admission status, requirement for mechanical ventilation, and laboratory parameters (albumin, hemoglobin, lymphocyte count, platelet count, neutrophil count, monocyte count, urea, creatinine, sodium, potassium, CRP, and INR). The primary endpoint, 90-day mortality, was ascertained as all-cause mortality by cross-referencing hospital medical records with the national death notification registry (MERNİS Death Notification System), and follow-up was complete for all 188 patients included in the final analysis.

### 2.3. Calculation of Inflammatory and Nutritional Indices

All indices investigated for their prognostic value in this study were calculated using hemogram and biochemistry parameters obtained from venous blood samples taken at the time of the patients’ initial admission to the emergency department. Calculations were based on g/L units for hemoglobin and albumin, and 10^9^/L units for cell counts (neutrophil, lymphocyte, monocyte, platelet). The indices were determined according to the following formulas:

- Systemic Immune-Inflammation Index (SII): Calculated by dividing the product of the neutrophil count and platelet count by the lymphocyte count (SII = (Neutrophil × Platelet)/Lymphocyte).
- Systemic Inflammation Response Index (SIRI): Calculated by dividing the product of the neutrophil count and monocyte count by the lymphocyte count (SIRI = (Neutrophil × Monocyte)/Lymphocyte).
- HALP Score: Defined as the ratio of the product of hemoglobin, albumin, and lymphocyte count to the platelet count (HALP = (Hemoglobin × Albumin × Lymphocyte)/Platelet).
- Modified HALP (m-HALP) Score: According to the method optimized by Kocaoglu et al., the product of hemoglobin, albumin, lymphocyte, and platelet values was taken, and the result was divided by 1000 to ensure clinical ease of use and readability (m-HALP = (Hemoglobin × Albumin × Lymphocyte × Platelet)/1000). It should be noted that division by 1000 is a linear scaling operation and therefore has no effect on rank-based discriminative performance metrics such as the AUC, sensitivity, or specificity; it serves solely to convert the raw product into a numerically more manageable range for clinical use. Notably, an independent large-database study of septic patients has also adopted the same 1000-fold scaling for identical readability purposes, indicating that this convention is not unique to the present study [17]. Physiologically, positioning platelet count as a multiplicative term rather than as a denominator, as in the conventional HALP score, aligns the score’s direction with the established prognostic role of thrombocytopenia in sepsis, which reflects consumptive coagulopathy, cytokine-mediated destruction, and marrow suppression, and is an independent marker of severity and mortality [18]. In the conventional formula, worsening thrombocytopenia paradoxically raises the score; in m-HALP it lowers the score, consistent with its other components.

All calculations were performed automatically and recorded in the database prior to the statistical analysis of the data.

### 2.4. Statistical Analysis

Post hoc power analysis was performed using G*Power 3.1. With a total of 188 patients (93 non-survivors, 95 survivors), the balanced distribution (~1:1) yielded a statistical power (1-β) of 92% at α = 0.05 level, assuming a medium effect size (d = 0.5). This exceeds the standard 80% threshold for statistical adequacy. Normality of the numeric data was tested with the Shapiro–Wilk test. Numerical variables are presented as mean ± standard deviation or median (minimum–maximum), for normally distributed or non-normal data, respectively. Categorical data were presented with frequencies (n) and percentages. Comparisons between two independent groups were performed using the independent samples *t*-test or Mann–Whitney U test, as appropriate. Comparisons between independent groups for categorical variables were performed with the Pearson χ^2^ test. Receiver operating characteristic (ROC) curve analysis was performed and area under the ROC curve (AUC) was estimated to examine the performance of indices in discriminating between 90-day survivors and non-survivors. Binary logistic regression analyses were performed to evaluate the independent association between m-HALP and 90-day mortality. For indices with significant performance, optimal cut-off values were calculated according to the Youden J index. *p* < 0.05 was accepted as statistically significant. Statistical analyses were performed with the IBM SPSS Statistics 29.0.0 software.

## 3. Results

The study included 188 patients with a median age of 75.50 (min-max: 30.00–101.00) years; 44.15% (n = 83) were male, 55.85% (n = 105) were female. 7-day mortality rate was 17.55% (n = 33), while the 90-day mortality rate was 49.47% (n = 93). Comparison of the clinical and laboratory characteristics of patients with and without 90-day mortality is given in Table 1.

Regarding the comparison of inflammatory indices based on 90-day mortality, only the m-HALP score was found to be statistically significantly lower in the non-survivor group (*p* = 0.033), whereas no significant differences were observed for HALP, SII, or SIRI scores between the groups (Table 2). In terms of short-term (7-day) mortality prediction, none of the evaluated indices (HALP, m-HALP, SII, or SIRI) demonstrated a statistically significant discrimination between survivors and non-survivors (*p* > 0.05) (Table 3). Regarding the distribution of infection sources, pneumosepsis was the most prevalent focus (44.7%), followed by urosepsis (27.1%) and cholangiosepsis (9.6%), respectively (Table 4).

**Figure 2:**
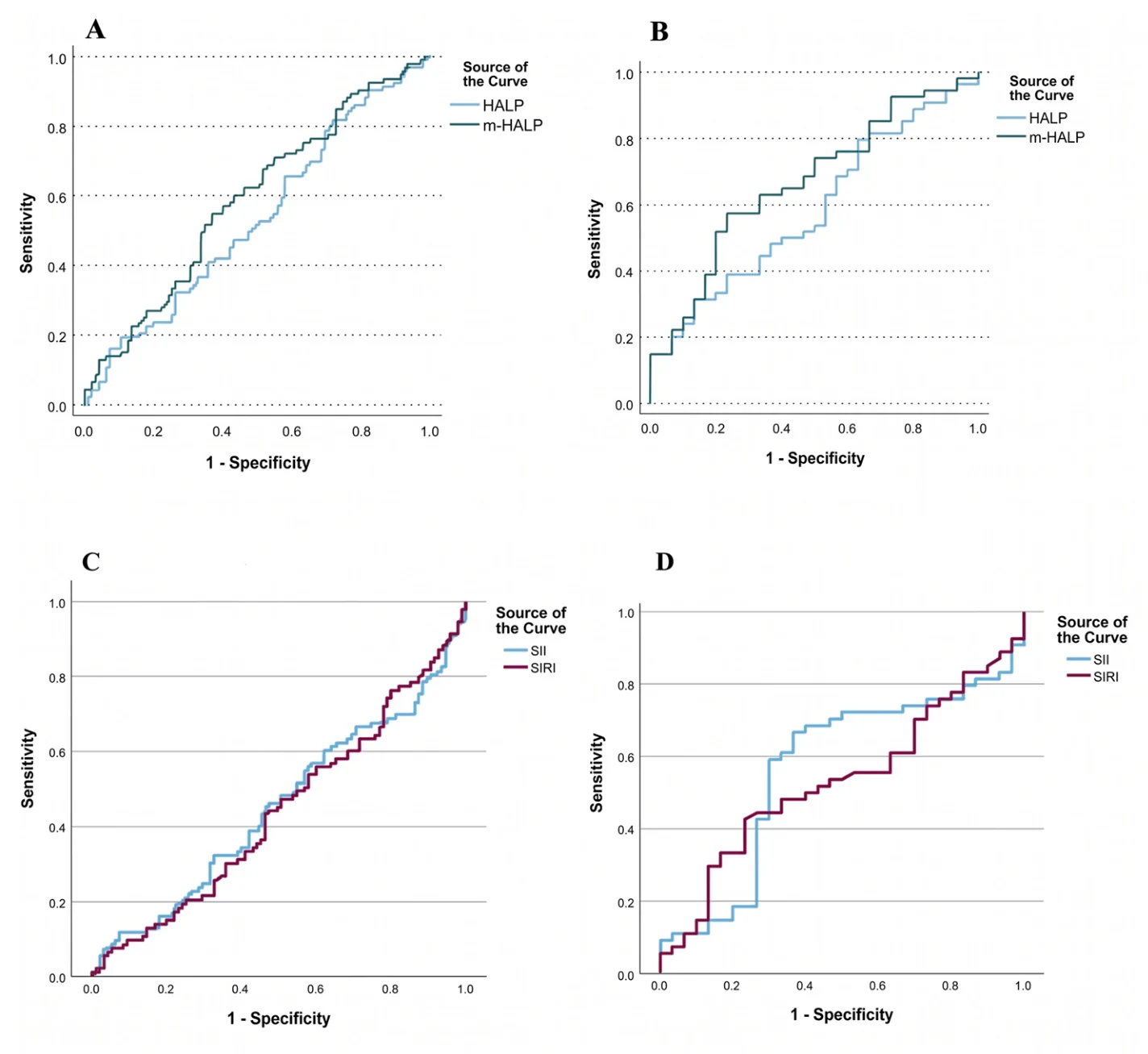
ROC curves predicting 90-day mortality based on: (**A**) HALP and m-HALP in all patients; (**B**) HALP and m-HALP in pneumosepsis patients; (**C**) SII and SIRI in all patients; (**D**) SII and SIRI in pneumosepsis patients.

The diagnostic performance of HALP, m-HALP, SII, and SIRI for predicting 90-day mortality was evaluated in the whole sample as well as in the pneumosepsis and urosepsis subgroups (Table 5). In the overall group, m-HALP demonstrated low but statistically significant discriminatory ability for 90-day mortality (AUC 0.590, 95% CI 0.509–0.671; *p* = 0.030), whereas HALP, SII, and SIRI did not show significant predictive performance (*p* = 0.410, *p* = 0.249, *p* = 0.158, respectively). At the optimal cut-off value of 522.11, m-HALP yielded a sensitivity of 0.55 and a specificity of 0.63 (positive predictive value (PPV) = 59.3%, negative predictive value (NPV) = 58.8%, positive likelihood ratio (LR+) = 1.49, negative likelihood ratio (LR−) = 0.72). Among patients with pneumosepsis, m-HALP gave a statistically significant AUC of 0.667 (95% CI: 0.548–0.785, *p* = 0.006). The optimal cut-off value was ≤522.11 again, corresponding to a sensitivity of 0.57 and a specificity of 0.77 (PPV = 81.6%, NPV = 50.0%, LR+ = 2.46, LR− = 0.56). HALP, SII, and SIRI did not demonstrate significant predictive value for 90-day mortality in patients with pneumosepsis. In the urosepsis subgroup, none of the evaluated indices, including HALP, m-HALP, SII, or SIRI, showed significant discrimination for 90-day mortality (Table 5, Figure 2). Based on the reported sensitivity, specificity, and outcome prevalence, the positive and negative predictive values and likelihood ratios of m-HALP were calculated as 59.3%, 58.8%, 1.49, and 0.72, respectively, in the overall cohort, and as 81.6%, 50.0%, 2.46, and 0.56, respectively, in the pneumosepsis subgroup; the higher positive predictive value observed in the pneumosepsis subgroup reflects the higher baseline mortality prevalence in this group rather than superior discriminative performance, as the prevalence-independent likelihood ratios remained well below the thresholds generally considered clinically meaningful in both analyses.

Based on the ROC-derived cut-off value of 522.11, m-HALP was categorized as low (≤522.11) or high (>522.11). Multivariable binary logistic regression analyses were subsequently performed to determine whether m-HALP was associated with 90-day mortality after adjustment for age, sex, and creatinine level. Separate models were constructed for the overall sample and the pneumosepsis subgroup. In the overall sample, patients with low m-HALP (≤522.11) had 1.91-fold higher odds of 90-day mortality than those with high m-HALP (>522.11) after adjustment for age, sex, and creatinine (OR = 1.91, 95% CI: 1.05–3.48, *p* = 0.034). Older age was also independently associated with increased odds of 90-day mortality (OR = 1.03, 95% CI: 1.01–1.05, *p* = 0.014). In the pneumosepsis subgroup, low m-HALP remained an independent predictor of 90-day mortality. Patients with low m-HALP (≤522.11) had 4.30-fold higher odds of 90-day mortality than those with high m-HALP (>522.11) after adjustment for age, sex, and creatinine (OR = 4.30, 95% CI: 1.54–12.02, *p* = 0.005), whereas age, sex, and creatinine were not independently associated with mortality (Table 6).

Secondary analyses were performed for 7-day mortality, ICU admission, and mechanical ventilation requirements (Table 7). None of the evaluated indices (HALP, m-HALP, SII, or SIRI) demonstrated statistically significant discriminatory performance for these endpoints (all *p* > 0.05). AUC values ranged between 0.416 and 0.555, indicating that these markers were not predictive of early mortality or immediate clinical severity in the study population.

## 4. Discussion

This study is among the first to evaluate the performance of novel inflammatory and nutritional indices in predicting short-term (7-day) and long-term (90-day) mortality in sepsis patients presenting to the emergency department. The primary finding of this study is that while traditional inflammatory indices (HALP, SII, SIRI) lacked prognostic value, the m-HALP score demonstrated a statistically significant, albeit modest, association with 90-day mortality. Although these findings suggest that integrating nutritional and immune parameters may offer theoretical advantages over purely inflammatory indices, the overall discriminatory performance of m-HALP remains preliminary and insufficient for standalone clinical application.

In our study, the m-HALP score was found to be associated with 90-day mortality in the overall sepsis cohort (*p* = 0.030), and this association became more pronounced in the pneumosepsis group, reaching an AUC value of 0.667. Unlike the classical HALP score, m-HALP offers a mathematical model that is more sensitive to changes in the platelet/lymphocyte balance while maintaining the weight of nutritional parameters such as hemoglobin and albumin, thanks to the product-based integration of the variables in the formula. It is not a coincidence that the m-HALP score, the prognostic value of which was previously demonstrated by Kocaoglu et al. in acute heart failure patients, also predicts long-term survival in a hypercatabolic process such as sepsis [16]. Sepsis can evolve beyond the acute phase into a chronic state of deterioration characterized by “Persistent Inflammation, Immunosuppression, and Catabolism Syndrome” (PICS). In this process, patient survival depends not only on the severity of the acute inflammatory response but also on the body’s metabolic reserves (albumin, hemoglobin). The fact that m-HALP predicted 90-day (long-term) mortality rather than 7-day (acute) mortality in our study is compatible with the hypothesis that this index reflects the patient’s long-term physiological reserve rather than acute physiological derangement. However, as no immunological or metabolic markers were directly measured in this study, this mechanistic interpretation remains speculative and requires confirmation in future studies.

Contrary to the general trend in the literature, the SII and SIRI indices failed to predict mortality in our study (*p* > 0.05). Some recent studies have reported that SII and SIRI are associated with mortality in sepsis and urosepsis patients [19,20,21]. This situation parallels some studies in the literature reporting “negative” results. For instance, Gurpinar et al. (2025) reported that SII and SIRI were not statistically significant in predicting mortality in patients with sepsis; similarly, Jiang et al. (2023) detected a non-linear (J-shaped) relationship between these indices and mortality, which may explain the ‘nonsignificant’ results in linear analyses [21,22]. Furthermore, Assinger et al. emphasized that sepsis-induced platelet dysfunction could affect the calculation of these scores, leading to deviations in prognostic accuracy [18].

The “negative” performance observed in our study may be explained by the demographic characteristics of our patient population. The median age of our study group was 75.5 years, which is higher than many studies in the literature (e.g., cohorts with a mean age of 60–65). Age-related immune dysfunction in the geriatric population causes a blunted neutrophil response and dysregulated lymphocyte apoptosis, thereby reducing the sensitivity of indices based on the neutrophil/lymphocyte ratio (SII, SIRI). Additionally, SII and SIRI are acute phase reactants and may change rapidly in the early period of infection; however, over a long period such as 90 days, as acute inflammation is replaced by comorbidities and nutritional depletion, the implications of these indices on long-term mortality diminish.

The standard HALP score also yielded non-significant results in our study (*p* = 0.410). However, studies by Li et al. and Zhang et al. reported that low HALP scores were associated with mortality [23,24]. The fundamental difference between our study and these studies lies in the mathematical optimization of the m-HALP formula. While the standard HALP formula [(Hb × Alb × Lymph)/Plt] may lead to deviations in patients with extreme platelet counts, m-HALP standardizes these parameters with a more balanced multiplier model [(Hb × Alb × Lymph × Plt)/1000]. The fact that m-HALP reached the highest prognostic accuracy (AUC: 0.667) in the pneumosepsis subgroup may reflect the role of protein and oxygen transport capacity (albumin and hemoglobin) in the repair of lung parenchymal injury.

A large-scale study based on the MIMIC-IV database examining the role of the m-HALP score in sepsis patients reported that this score was associated with 30-day mortality in intensive care patients [17]. While such large database studies offer robust statistical power for multivariable modeling, they primarily reflect data from the intensive care setting after initial resuscitation. Our study attempts to explore a different clinical niche by evaluating the “Time 0” physiological snapshot at the moment of admission to the emergency department, focusing on 90-day long-term mortality rather than short-term outcomes. While our study found a statistically significant association with 90-day mortality, the poor-to-modest AUC values clearly indicate that m-HALP lacks sufficient predictive power to be used as a standalone prognostic triage tool. Notably, multivariable analysis confirmed that this association was independent of age, sex, and renal function, indicating that m-HALP’s prognostic signal is not merely a proxy for these established risk factors. However, independent statistical association does not equate to adequate individual-level discriminative accuracy, and the modest AUC values indicate that m-HALP still cannot reliably classify individual patients’ 90-day mortality risk on its own. Furthermore, although the discriminative ability was relatively higher in the pneumosepsis subgroup (AUC 0.667), this finding must be interpreted with caution due to the small subgroup sample size and the inherent risks of multiple testing. Therefore, the distinctive association observed provides complementary, yet strictly exploratory and hypothesis-generating evidence regarding the pathophysiological role of this index in reflecting the patient’s long-term metabolic reserve during hypercatabolic states, rather than serving as an actionable clinical threshold.

### Strengths and Limitations of the Study

The primary strengths of the study include high clinical applicability provided by the use of routine parameters, the evaluation of 90-day long-term mortality, and subgroup analyses specific to the source of infection (pneumosepsis/urosepsis). However, the risk of selection bias inherent to the single-center and retrospective design, along with the lack of serial measurements (dynamic trends), weakens the causal relationship. Additionally, the small sample size in other infection sources limits the generalizability of the study, and the added contribution of the indices to clinical scores has not been fully verified with calibration or decision curve analyses (DCA). Furthermore, due to the retrospective nature of the study, serum lactate levels and arterial blood gas parameters required for SOFA scores were not routinely available at initial presentation. Excluding patients without these data would have drastically reduced the sample size and exacerbated selection bias; thus, they could not be included for direct comparison. It is recommended that future prospective studies investigate the dynamic changes in m-HALP with serial measurements, validate its independent prognostic value in larger, multicenter multivariable cohorts, and its external validity in different populations. Furthermore, this study involved a large number of comparisons across four indices, four clinical outcomes, and three patient subgroups, and no correction for multiple testing was applied; the single positive finding should therefore be interpreted with caution and considered hypothesis-generating rather than confirmatory. ICU admission was included as a secondary outcome because it reflects the clinical escalation of care in the immediate post-admission period; however, this endpoint is strongly influenced by institutional practice patterns, bed availability, and individual physician judgment rather than by disease severity alone. Albumin, ICU admission, and mechanical ventilation status were not incorporated as covariates in the multivariable model: albumin is a direct component of the m-HALP formula and its inclusion would introduce structural collinearity, whereas ICU admission and mechanical ventilation are concurrent or downstream clinical events rather than baseline confounders. Nonetheless, residual confounding by unmeasured or unadjusted severity-related factors cannot be fully excluded and remains a limitation of this analysis.

## 5. Conclusions

In conclusion, although the m-HALP score demonstrated a statistically significant association with 90-day mortality compared to the standard HALP, SII, and SIRI indices, its overall discriminatory performance remains poor-to-modest in sepsis patients presenting to the emergency department. This low diagnostic accuracy underscores that statistical significance does not equate to clinical utility, rendering m-HALP inadequate for standalone bedside decision-making. The relatively better performance observed in the pneumosepsis subgroup suggests that integrating both the inflammatory response and nutritional reserve may offer valuable pathophysiological insights; nevertheless, this finding is purely hypothesis-generating. At this stage, m-HALP should be considered an exploratory biomarker rather than a definitive clinical tool for long-term risk stratification. Further prospective, multicenter studies are required to validate these findings and confirm the independent prognostic value of m-HALP identified in the present multivariable analysis.

## Figures and Tables

**Table 1:** Clinical and laboratory characteristics and comparison of patients with and without 90-day mortality.

Variable	Total (n = 188)	90-Day Mortality		*p*-Value *
		Survival (n = 95)	Non-Survival (n = 93)	
Age (years), M (Min–Max)	75.50 (30.00–101.00)	73.00 (30.00–101.00)	78.00 (45.00–98.00)	**0.013**
Sex (Male), n (%)	83 (44.15%)	43 (45.26%)	40 (43.01%)	0.756
ICU admission, n (%)	161 (85.64%)	73 (76.84%)	88 (94.62%)	**<0.001**
Mechanical ventilation, n (%)	47 (25.00%)	4 (4.21%)	43 (46.24%)	**<0.001**
Albumin (g/L), Mean ± SD	29.06 ± 6.28	30.24 ± 6.22	27.85 ± 6.13	**0.009**
Hemoglobin (g/L), Mean ± SD	109.94 ± 25.61	112.65 ± 23.24	107.16 ± 27.67	0.143
Lymphocyte count, M (Min–Max)	0.80 (0.10–10.10)	0.80 (0.10–10.10)	0.70 (0.10–6.10)	0.261
Platelet count, M (Min–Max)	221.50 (6.00–965.00)	222.00 (32.00–965.00)	220.00 (6.00–678.00)	0.161
Neutrophil count, M (Min–Max)	11.50 (1.00–77.30)	11.80 (2.70–31.80)	10.30 (1.00–77.30)	0.196
Monocyte count, M (Min–Max)	0.60 (0.10–3.60)	0.80 (0.10–3.60)	0.60 (0.10–3.10)	0.050
Urea (mg/dL), M (Min–Max)	92.50 (5.60–409.00)	73.00 (5.60–409.00)	106.00 (17.0–386.0)	**0.046**
Creatinine (mg/dL), M (Min–Max)	1.71 (0.36–9.19)	1.67 (0.54–8.63)	1.77 (0.36–9.19)	**0.005**
Sodium (mmol/L), M (Min–Max)	134.00 (110.0–180.0)	133.00 (110.0–165.0)	135.00 (115.0–180.0)	0.176
Potassium (mmol/L), M (Min–Max)	4.20 (2.30–8.20)	4.10 (2.30–8.20)	4.30 (2.40–8.20)	0.188
CRP (mg/L), M (Min–Max)	152.50 (3.00–214.00)	153.00 (3.30–212.00)	140.00 (3.00–214.00)	0.874
INR, M (Min–Max)	1.27 (0.94–23.12)	1.25 (0.95–23.12)	1.28 (0.94–19.46)	0.690

Continuous variables are presented as mean ± standard deviation or median (minimum–maximum), as appropriate. Categorical variables are presented as n (%). M: Median, Min–Max: minimum–maximum. * Comparison of patients with and without 90-day mortality. Values in bold indicate statistical significance (*p* < 0.05).

**Table 2:** Inflammatory indices and comparison of inflammatory indices according to 90-day mortality.

Variable	Total (n = 188)	90-Day Mortality		*p*-Value *
		Survival (n = 95)	Non-Survival (n = 93)	
HALP score	11.32 (0.97–123.47)	11.41 (0.97–123.47)	11.25 (1.24–110.19)	0.411
m-HALP score	578.29 (39.00–11,250.07)	677.24 (24.96–11,250.07)	485.68 (3.90–6777.34)	**0.033**
SII	3540.00 (6.0–28,272.0)	3745.33 (292.85–27,300.0)	3142.00 (6.00–28,272.0)	0.250
SIRI	7.24 (1.00–218.50)	7.84 (1.94–206.70)	6.64 (1.00–218.50)	0.161

(m-)HALP: (modified) hemoglobin, albumin, lymphocyte, and platelet score. SII: Systemic Immune-Inflammation Index. SIRI: Systemic Inflammation Response Index. Data given as median (min–max) values. * Comparison of patients with and without 90-day mortality. Values in bold indicate statistical significance (*p* < 0.05).

**Table 3:** Comparison of inflammatory indices according to 7-day mortality.

Variable	7-Day Mortality		*p*-Value
	Survival (n = 155)	Non-Survival (n = 33)	
HALP score	11.25 (0.97–123.47)	12.38 (1.27–110.19)	0.381
m-HALP score	604.94 (4.00–11,250.07)	518.66 (0.39–6777.34)	0.395
SII	3745.33 (161.50–28,272.00)	2800.00 (6.00–19,710.00)	0.131
SIRI	6.95 (0.19–218.50)	7.89 (0.10–148.65)	0.773

(m-)HALP: (modified) hemoglobin, albumin, lymphocyte, and platelet score. SII: Systemic Immune-Inflammation Index. SIRI: Systemic Inflammation Response Index. Data given as median (min–max) values.

**Table 4:** Distribution of patients according to source of infection.

Source of Infection	n (%)
Pneumosepsis	84 (44.7)
Urosepsis	51 (27.1)
Cholangiosepsis	18 (9.6)
Cellulitis	8 (4.3)
Gastroenteritis	7 (3.7)
Peritonitis	4 (2.1)
Encephalitis	2 (1.1)
Soft tissue infection	2 (1.1)
Pancreatitis	2 (1.1)
Intra-abdominal abscess	2 (1.1)
Meningitis	2 (1.1)
Pressure ulcer infection	1 (0.5)
Infective endocarditis	1 (0.5)
Decubitus ulcer infection	1 (0.5)
Gas gangrene	1 (0.5)
Necrotizing fasciitis	1 (0.5)
Cerebral hydatid cyst infection	1 (0.5)
Total	188 (100.0)

**Table 5:** Diagnostic performance of inflammatory and nutritional indices for 90-day mortality across patient subgroups.

Patient Group	Index	AUC (95% CI)	*p*-Value	Cut-Off	Sensitivity	Specificity
All patients	HALP	0.535 (0.452–0.617)	0.410	—	—	—
	m-HALP	0.590 (0.509–0.671)	**0.030**	522.11	0.548	0.632
	SII	0.451 (0.369–0.534)	0.249	—	—	—
	SIRI	0.441 (0.359–0.523)	0.158	—	—	—
Pneumosepsis	HALP	0.585 (0.460–0.709)	0.184	—	—	—
	m-HALP	0.667 (0.548–0.785)	**0.006**	522.11	0.574	0.767
	SII	0.567 (0.436–0.697)	0.316	—	—	—
	SIRI	0.532 (0.407–0.657)	0.618	—	—	—
Urosepsis	HALP	0.480 (0.318–0.641)	0.805	—	—	—
	m-HALP	0.511 (0.344–0.678)	0.897	—	—	—
	SII	0.371 (0.208–0.535)	0.123	—	—	—
	SIRI	0.373 (0.214–0.533)	0.119	—	—	—

AUC: area under the receiver operating characteristic curve; CI: confidence interval. Values in bold indicate statistical significance (*p* < 0.05).

**Table 6:** Binary logistic regression analysis for 90-day mortality in the overall cohort and pneumosepsis subgroup.

Variables	Overall Sample		Pneumosepsis Subgroup	
	OR (95% CI)	*p*-Value	OR (95% CI)	*p*-Value
m-HALP	1.91 (1.05–3.48)	**0.034**	4.30 (1.54–12.02)	**0.005**
Age	1.03 (1.01–1.05)	**0.014**	1.01 (0.97–1.05)	0.811
Sex	1.18 (0.65–2.15)	0.591	0.97 (0.37–2.51)	0.950
Creatinine	1.08 (0.90–1.29)	0.398	1.03 (0.75–1.41)	0.845

Overall: Omnibus *p* = 0.011; Hosmer–Lemeshow *p* = 0.759. Pneumosepsis subgroup: Omnibus *p* = 0.049; Hosmer–Lemeshow *p* = 0.335. Reference category for m-HALP: >522.11. Reference category for sex: male. OR: odds ratio; CI: confidence interval. Values in bold indicate statistical significance (*p* < 0.05).

**Table 7:** Diagnostic performance of inflammatory and nutritional indices for 7-day mortality, ICU admission, and need for mechanical ventilation.

Variables	Index	AUC (95% CI)	*p*-Value
7-day mortality	HALP	0.451 (0.341–0.562)	0.389
	m-HALP	0.547 (0.433–0.661)	0.416
	SII	0.416 (0.306–0.526)	0.135
	SIRI	0.484 (0.370–0.598)	0.783
ICU admission	HALP	0.456 (0.345–0.568)	0.445
	m-HALP	0.555 (0.437–0.674)	0.360
	SII	0.441 (0.325–0.556)	0.315
	SIRI	0.440 (0.320–0.560)	0.324
Mechanical ventilation	HALP	0.516 (0.422–0.611)	0.735
	m-HALP	0.529 (0.426–0.632)	0.582
	SII	0.476 (0.378–0.575)	0.638
	SIRI	0.481 (0.385–0.577)	0.697

AUC: area under the receiver operating characteristic curve; CI: confidence interval. AUC values below 0.5 were not inverted, as they were close to 0.5 and not statistically significant, and therefore did not indicate meaningful discriminatory performance.

## Data Availability

The datasets used and/or analyzed during the current study are available from the corresponding author upon reasonable request.

## Abbreviations

SII	Systemic Immune-Inflammation Index
SIRI	Systemic Inflammation Response Index
m-HALP	Modified Hemoglobin, Albumin, Lymphocyte, and Platelet score
HALP	Hemoglobin, Albumin, Lymphocyte, and Platelet score
ICU	Intensive Care Unit
ROC	Receiver Operating Characteristic
AUC	Area Under the Curve
CI	Confidence Interval
CRP	C-reactive Protein
PCT	Procalcitonin
SOFA	Sequential Organ Failure Assessment
qSOFA	quick Sequential Organ Failure Assessment
INR	International Normalized Ratio
PICS	Persistent Inflammation, Immunosuppression, and Catabolism Syndrome
DCA	Decision Curve Analyses
